# Different Approaches for Extracting Information from the Co-Occurrence Matrix

**DOI:** 10.1371/journal.pone.0083554

**Published:** 2013-12-26

**Authors:** Loris Nanni, Sheryl Brahnam, Stefano Ghidoni, Emanuele Menegatti, Tonya Barrier

**Affiliations:** 1 Department of Information Engineering, University of Padua, Padova, Italy; 2 Department of Computer Information Systems, Missouri State University, Springfield, Missouri, United States of America; Institute of Psychology, Chinese Academy of Sciences, China

## Abstract

In 1979 Haralick famously introduced a method for analyzing the texture of an image: a set of statistics extracted from the co-occurrence matrix. In this paper we investigate novel sets of texture descriptors extracted from the co-occurrence matrix; in addition, we compare and combine different strategies for extending these descriptors. The following approaches are compared: the standard approach proposed by Haralick, two methods that consider the co-occurrence matrix as a three-dimensional shape, a gray-level run-length set of features and the direct use of the co-occurrence matrix projected onto a lower dimensional subspace by principal component analysis. Texture descriptors are extracted from the co-occurrence matrix evaluated at multiple scales. Moreover, the descriptors are extracted not only from the entire co-occurrence matrix but also from subwindows. The resulting texture descriptors are used to train a support vector machine and ensembles. Results show that our novel extraction methods improve the performance of standard methods. We validate our approach across six medical datasets representing different image classification problems using the Wilcoxon signed rank test. The source code used for the approaches tested in this paper will be available at: http://www.dei.unipd.it/wdyn/?IDsezione=3314&IDgruppo_pass=124&preview=.

## Introduction

When it comes to data, we live in unprecedented times. Across industries and academia, companies and institutions are struggling to handle the massive amounts of data that are accumulating daily. This explosion of data is coming from multiple sources: sensors, social media, the Internet, smart devices, and smart phones. Data stockpiling is motivated in large part by the availability of inexpensive data storage and commodity computer systems that have nearly the computational power of earlier supercomputers [1]. These advances in computing and the economics of ownership have greatly accelerated research in technologies that are producing even more raw data. This is especially the case with computer vision [2] [3] [4], which is now a major component in many applications, ranging from video surveillance software to robotic systems and automatic visual inspection systems for checking industrial products at the end of the production line.

Medical imaging, in particular, is one field that is witnessing rapid technological growth along with a concomitant avalanche of data. To handle this data, specialized research databases (e.g., HUGO, Rfam, and Cancer Cell Map) and metadatabases (e.g., Biograph, mGen, PathogenPortal, and ConsensusPathDB) have been established. Machine vision technology applied to many of these databases has the potential of revolutionizing scientific knowledge in medicine. Already some invaluable gains have been made in the detection of tumors and cancers. For example, in [5] image texture information is utilized to automatically discriminate polyps in colonoscopy images, and in [4] linear discriminant analysis of wavelet features, which has been successfully employed in many nonmedical applications (for instance, traffic accident detection [6] and face identification and verification [7]), is proving highly effective in the detection of tumors in endoscopic images.

Texture analysis is often involved in image classification, but there is no universally recognized definition of texture. It can be viewed as a global pattern arising from the repetition of local subpatterns [8] or as a region where a set of local properties or statistics are either constant, slowly varying, or approximately periodic [9] (for an interesting early catalogue of definitions see [10]). Many different methods for managing texture have been developed that are based on the various ways texture can be characterized. Some of the highest performing methods reported in the literature include the scale-invariant feature transform (SIFT) [11], speeded up robust feature (SURF) [12], histogram of oriented gradients (HOG) [13], gradient location and orientation histogram (GLOH) [14], region covariance matrix (RCM) [15], edgelet [16], gray level co-occurrence matrix (GLCM) [17], local binary patterns (LBP) [18], nonbinary encodings [19], color correlogram (CCG) [20], color coherence vectors (CCV) [21], color indexing [22], steerable filters [23] and Gabor filters [24].

Arguably the Local Binary Pattern (LBP) operator [25] is one of the most powerful approaches utilizing the texture information in an image. LBP is simple, effective, and robust and is proving to be a powerful discriminator in many medical image classification problems. In [26], for example, LBP assigns a Marsh-like score to endoscopical images of pediatric celiac diseases. In [27] a Support Vector Machine (SVM) is coupled with the LBP operator to distinguish real masses from normal parenchyma in mammographic images. LBP has also been combined with other descriptors useful for medical data mining purposes. In [28], for instance, LBP is used to explore brain magnetic resonance data, and in [29] the authors demonstrate how a combination of LBP with other texture descriptors is effective in classifying different cell phenotypes.

In this paper, we focus on improving one of the earliest methods for analyzing texture: the GLCM, originally proposed by Haralick [30] in 1979 for analyzing satellite images. Based on a set of features, or descriptors, that are evaluated starting from a histogram, GLCM is one of the most studied and extensively used general approaches for texture analysis and has recently become the focus of several research groups whose aim is to increase the discriminability of GLCM descriptors. Some interesting recent work in this area includes [31], where the authors consider different values of the distance parameter that influences the GLCM. In [32] features are extracted by weighted summation of GLCM elements from areas presenting high discrimination. In [33] descriptors are obtained from the gray level-gradient co-occurrence matrix, formed by calculating the gradient value of each pixel in the neighborhood of interest points. In [34] GLCM is combined with the edge orientation co-occurrence matrix of superior order [35], thereby taking into consideration both the gray levels of the image pixels and such local features as edges. Multi-scale analysis has also been performed using the GLCM. For example, in [36] and in [37] multiple scales are considered by changing the window size used to extract the GLCM descriptors, and in [38] the image is rescaled to different sizes and co-occurrence descriptors are extracted from each rescaling.

Recently the addition of color information has been evaluated for co-occurrence matrices [39]. In [40], for instance, a colors-gradient co-occurrence matrix (CGCM) is proposed from which 27-dimensional statistical features are extracted. In [41] superior results are obtained on content-based image retrieval by employing a combination of the contourlet transform and the GLCM. First, the contourlet transform is performed for four subbands of the image, and then GLCM features are extracted from each band. In [42] the authors propose the Gradient Magnitude based Angle Co- occurrence Matrix for color image classification that is based on three different types of gradients defined in the RGB space.

Finally, some very recent work has been proposed that combines LBP with GLCM. For example, in [43], GLCM is constructed after LBP is applied to the image. Features are then extracted from second-order statistical parameters of the resulting LBGLCM matrix. Similarly, in [44], an LBP image is built after performing a Gaussian filtering pyramid preprocessing step, and the GLCM is constructed from the resulting LBP image.

A major difficulty encountered when analyzing texture is that results strongly depend on image resolution and scale, an effect that is especially problematic with edge-based approaches. One of the goals of this work is to assess the performance improvement that can be gained using a multi-scale approach. In the literature, several authors have recently reported combining several multi-scale approaches with LBP descriptors (see, for instance, [45] and [46]). We show in this study that it is also possible to improve the performance of different descriptors extracted from the co-occurrence matrix by coupling them with a multi-scale approach.

Our main intention, however, is to compare different methods for extracting features given the co-occurrence matrix (we are not comparing and combining different pre-processing methods applied before the co-occurrence matrix extraction). As described in detail in section 2, the following extraction methods are investigated:

- Extracting descriptors using the standard approach proposed by Haralick [17];
- Extracting a set of 3D descriptors by considering the co-occurrence matrix as a 3D shape [47];
- Extracting descriptors from different 2D shapes by considering the co-occurrence matrix as a 3D shape: The 2D shapes are obtained by intersecting the co-occurrence matrix with a set of horizontal planes at given heights;
- Extracting gray-level run-length features [48];
- Directly using the co-occurrence matrix as a descriptor by projecting it onto a lower dimensional subspace using principal component analysis;

We improve the performance of these descriptors further by extracting them not only from the entire original co-occurrence matrix, but also from multiple scales of the original image obtained by Gaussian filtering and by dividing the original co-occurrence matrix into different subwindows and extracting features separately from each subwindow. These descriptors are then used to train separate SVM classifiers whose results are combined by sum rule or weighted sum rule.

The experimental results presented in section 4 show that our new approach improves the performance of standard methods and some state-of-the-art approaches. We validate our approach across six medical datasets, described in section 3, representing very different image classification problems.

### Proposed System

The focus of this paper is on extracting descriptors from the co-occurrence matrix with the goal of enhancing the performance of Haralick’s descriptors. Improvements are achieved by applying the multi-scale approach that overcomes the main weakness of texture-based features (i.e., the dependency on scale discussed in the introduction) and by extracting features from different subwindows of the co-occurrence matrix and then combining them.

Each of the approaches tested in the experimental section is explained below. In all experiments, SVM [49]–[51] is used as the base classifier. In this study, linear, polynomial and radial basis function kernels are tested. For each dataset, the best kernel and the best set of parameters are chosen using a 5-fold cross validation approach on the training data.

### The Multi-scale Approach

**Figure 1:**
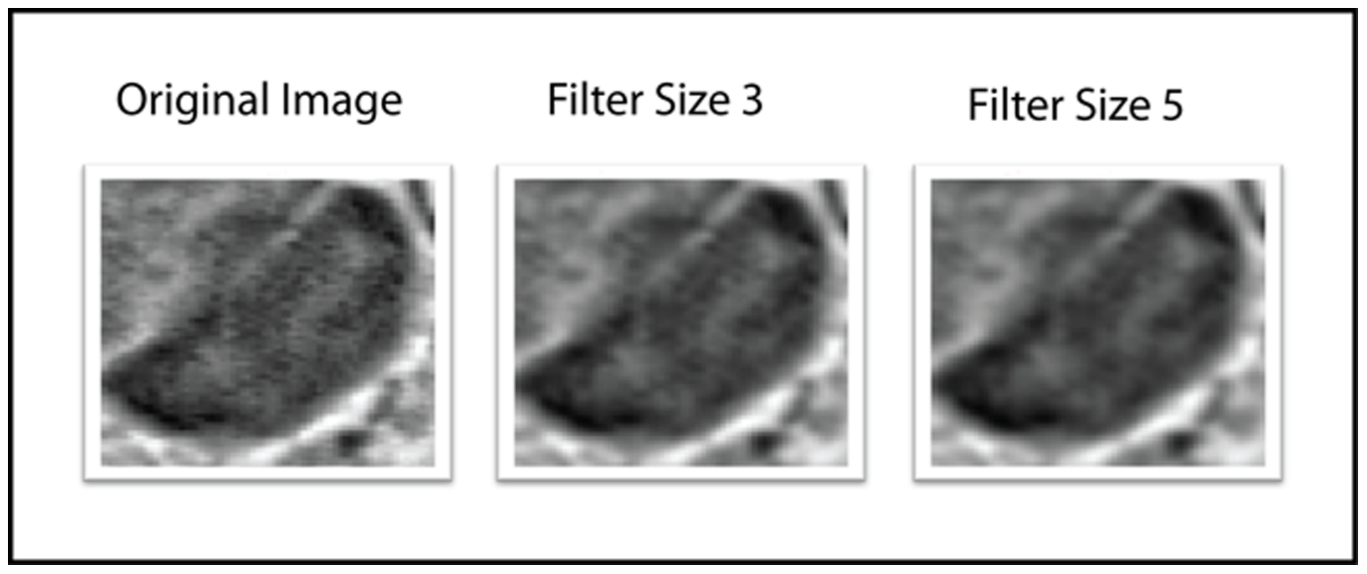
Images illustrating the effect of the multi-scale approach.

Using this approach, images are generated by applying a 2D symmetric Gaussian lowpass filter of size *k* (we use *k* = 3 and *k* = 5) with standard deviation 1. As illustrated in Figure 1, the original image is filtered to obtain a set of smoothed versions of the original image.

### The GLDM Co-occurrence Matrix

GLDM [17], [30] is a particular type of co-occurrence matrix obtained as the histogram on a 2D domain of dimension N_GL_×N_GL_, where N_GL_ is the number of gray levels in the image (typically 256). In other words, the co-occurrence matrix counts the number of gray level transitions between two pixel values such that the bin of the histogram whose coordinates are equal to the values of the two pixels is incremented. The way pixel couples are determined depends on the two parameters, *d* and *θ*. A value of *d* = 1 and *θ* = 0, for example, would produce pixel couples that are adjacent to each other on the same row. In our experiments four directions are considered: the horizontal (H), the vertical (V), the diagonal top left-bottom right, or right-down (RD), and the top right-bottom left, or left-down (LD).

### The Standard Haralick Statistics

The idea of using statistical indicators was originally proposed in [30]. In the experimental section, we label this approach HAR. The following HAR indicators are evaluated:

1. Energy
2. Correlation
3. Inertia
4. Entropy
5. Inverse difference moment
6. Sum average
7. Sum variance
8. Sum entropy
9. Difference average
10. Difference variance
11. Difference entropy
12. Information measure of correlation 1
13. Information measure of correlation 2.

A set of 13 descriptors is calculated from each co-occurrence matrix evaluated at *θ* = {0°, 45°, 90°, 135°} and with distance *d* = {1, 3}. A descriptor is obtained by concatenating the features extracted for each distance and orientation value.

In our experimental section, we also report the performance of several HAR variants:

- **HR**: features are extracted from the whole image only (note: several comparisons of different parameters settings for HR are reported in [47], but in this paper we use only the best configuration reported in that study).
- **HRsub**: a feature set is extracted from the whole co-occurrence matrix and from each subwindow (we use four subwindows in this work). Each set of features is then used to train a separate SVM. All 5 SVMs are combined by weighted sum rule, with weight of 4 for the SVM trained on the whole matrix, and weight of 1 for the others fours SVMs. To avoid presenting huge table, only the results of the best four subwindows are presented, defined using the coordinates (0, 0) to (127, 127); (128, 128) to (255, 255); (0, 128) to (255, 128); and (0, 128) to (128, 255);
- **HRsca,** features are extracted from the original image and the two filtered images The 15 SVMs are combined by weighted sum rule, with weight of the SVMs trained using features extracted from the original image given the value of 4, while the other SVMs have a weight of 1.

### SHAPE

**Figure 2:**
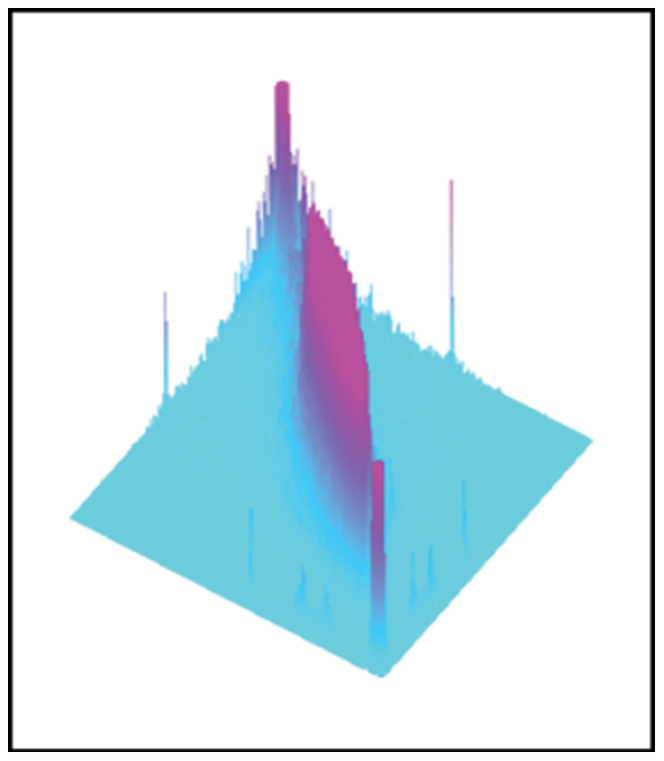
Illustration of the co-occurrence matrix as a 3D function.

The SHAPE approach explores the shape of the co-occurrence matrix by considering it as a 3D function (see Figure 2). This approach has been explored in detail in [52], [47], and [53]. The main idea of SHAPE is to intersect the GLDM with a set of horizontal planes at given heights and then derive a set of features based on the contours of the intersection, which defines a complex shape made up of one or more extractable blobs. The blob with the largest area, referred to as the main blob, is selected for extracting features and is fitted to an ellipse in order to simplify analysis. Although this approximation of the main blob shape to an ellipse results in some information loss, it offers the advantage of making the comparison among curves much easier.

Level curves are considered towards the base of the co-occurrence matrix, starting at height 1 and then going until height 19, with a distance of 2 between two consecutive planes. Level curves are all at a relatively low height because that region is very stable unlike the upper part of the co-occurrence matrix, which is much more unstable because of image noise. For this reason the co-occurrence matrix is not normalized, since normalization to the highest bin would introduce instabilities. Other types of normalization could be performed with respect to the total volume of the co-occurrence matrix, but this depends on the size of the original image, which is constant in most cases, making the normalization useless.

For each level, a set of descriptors extracted from the ellipses derived from the co-occurrence matrix is evaluated. The features describing all levels are then jointly analyzed for extracting a set of nine features that describe the evolution of the level curves (see [47], [52] for details).

These features are used to provide a characterization of the input image and can be directly used as input for a classifier. This is the principle exploited in the HAR approach. In the case of the SHAPE approach, however, features are evaluated not only on the entire co-occurrence matrix (as in [52]) but also on 12 subwindows of the GLDM defined by the following coordinates: #1: (0, 0) to (127, 127); #2: (128, 128) to (255, 255); #3: (0, 0) to (191, 191); #4: (64, 64) to (255, 255); #5: (0, 0) to (95, 95); #6: (31, 31) to (95, 95); #7: (63, 63) to (127, 127); #8: (95, 95) to (159, 159); #9: (127, 127) to (191, 191); #10: (159, 159) to (223, 223); #11: (191, 191) to (255, 255); and #12: (63, 63) to (191, 191). Several experiments using the entire GLDM along with these same subwindows are reported in [47]. (Note: in the case of SHAPE, we use more than the four subwindows because the performance of SHAPE improves when smaller subwindows are used; this is not the case with the other methods explored in this work).

For each of these 13 windows (counting the GLMD as a whole along with the 12 subwindows) a different feature vector is extracted, and these 13 descriptors are used to train 13 separate SVMs. Results are combined by weighted sum rule, where a weight of 1 is assigned to the first five descriptors, and a weight of 0.5 is assigned to the remainder. Each set of 13 descriptors is derived from co-occurrence matrices evaluated at *θ* = {0°, 45°, 90°, 135°} and, in the case when multiple values are used for the distance, *d* = {1, 3}. The feature vector is obtained by concatenating the features extracted for each value of the distance. In the experimental section, *SH* refers to the case where features are extracted from the entire co-occurrence matrix only, while *SHsub* is the method based on all 13 windows. When *SHsub* is coupled with the multi-scale approach, we call this combination *SHsca.*

### Curvature (CR)

The curvature (CR) algorithm [54] takes as input a set of edge point samples and a set of circular masks with varying radii. CR counts the number of samples falling in each mask when the mask is centered on edge points representing a contour. This count is used to obtain a measure of curvature, and these curvature measures are then quantized in a histogram of bins of equal size which are collected to form a feature vector.

The CR algorithm is applied to the co-occurrence matrix considering a set of level curves starting at height 1, going until height 15, with a distance of 3 between two consecutive planes. For each 2D shape defined for a given height, the descriptors are extracted from co-occurrence matrices evaluated at *θ* = {0°, 45°, 90°, 135°}, and multiple values of *d* = {1, 3}. The descriptor is obtained by concatenating the features extracted for each value of the distance and of the orientation. In the experimental section, we also report the performance of the following GL variants:

- **CU**: as in HR but using the CR features;
- **CUsub**: as in HRsub but using the CR features;
- **CUsca**: as in HRsca but using the CR features.

### 1.5 Gray-level Run-length Features (GL)

GL [48] derives descriptors from a run-length matrix that is based on characteristics of the gray level runs within a given image. A gray level run is a set of consecutive pixels with the same value, and the run length is the number of pixels in the set. The run-length matrix ***P*** contains in each location *p*(*i*, *j*) the number of runs of length *j* at a given gray level *i.* Starting from the run-length matrix it is possible to obtain several indicators, as explained in [48]. In our experiments, we consider the following:

- Short Run Emphasis (SRE), evaluated as:

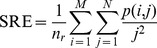
where 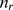 is the total number of runs, 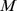 the number of gray levels, and 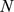 the maximum run length.

- Long Run Emphasis (LRE):

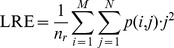

- Gray Level Nonuniformity (GLN):

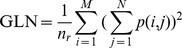

- Run Length Nonniformity (RLN):

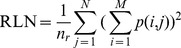

- Run Percentage (RP):

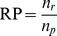
where 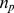 is the total number of pixels in the image.

- Low Grey-Level Run Emphasis (LGRE):

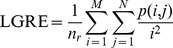

- High Grey-Level Run Emphasis (HGRE):

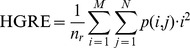

- Short Run Low Grey Level Emphasis (SRLGE):

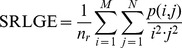

- Short Run High Grey Level Emphasis (SRHGE):

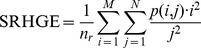

- Long Run Low Grey Level Emphasis (LRLGE):

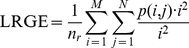

- Long Run High Grey Level Emphasis (LRHGE):

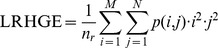



In our system we calculate the indicators described above from a run-length matrix that is evaluated on the GLDM. Since multiple GLDMs are calculated at several values of *θ* = {0°, 45°, 90°, 135°} and several values of *d* = {1, 3}, the GL descriptor is obtained by concatenating all the described features for all values of *θ* and d. The GL approach in turn has its own orientation: all values considered in [48] are evaluated in our system, namely *θ_GL_* = {0°, 45°, 90°, 135°}.

In the experimental section, we also report the performance of the following GL variants:

- **GR**: as in HR but using the GL features;
- **GRsub**: as in HRsub but using the GL features;
- **GRsca**: as in HRsca but using the GL features.

### Lower Dimensional Subspace (LDS)

In our experiments, we also use the co-occurrence matrix itself as a descriptor. Since it is very high in dimensionality, we project it onto a lower dimensional subspace using PCA, where 99% of the variance is retained for input into an SVM. As in the previous approaches, each set of features is extracted from co-occurrence matrices evaluated at *θ* = {0°, 45°, 90°, 135°}, and multiple values are used for the distance, *d* = {1, 3}. The descriptor is obtained by concatenating the features extracted for each value of the distance and of the orientation.

The use the following approaches with LDS:

1. **LD**: as in HR but using the LDS features;
2. **LDsub**: as in HRsub but using the LDS features;
3. **LDsca**: as in HRsca but using the LDS features.

## Datasets

**Figure 3:**
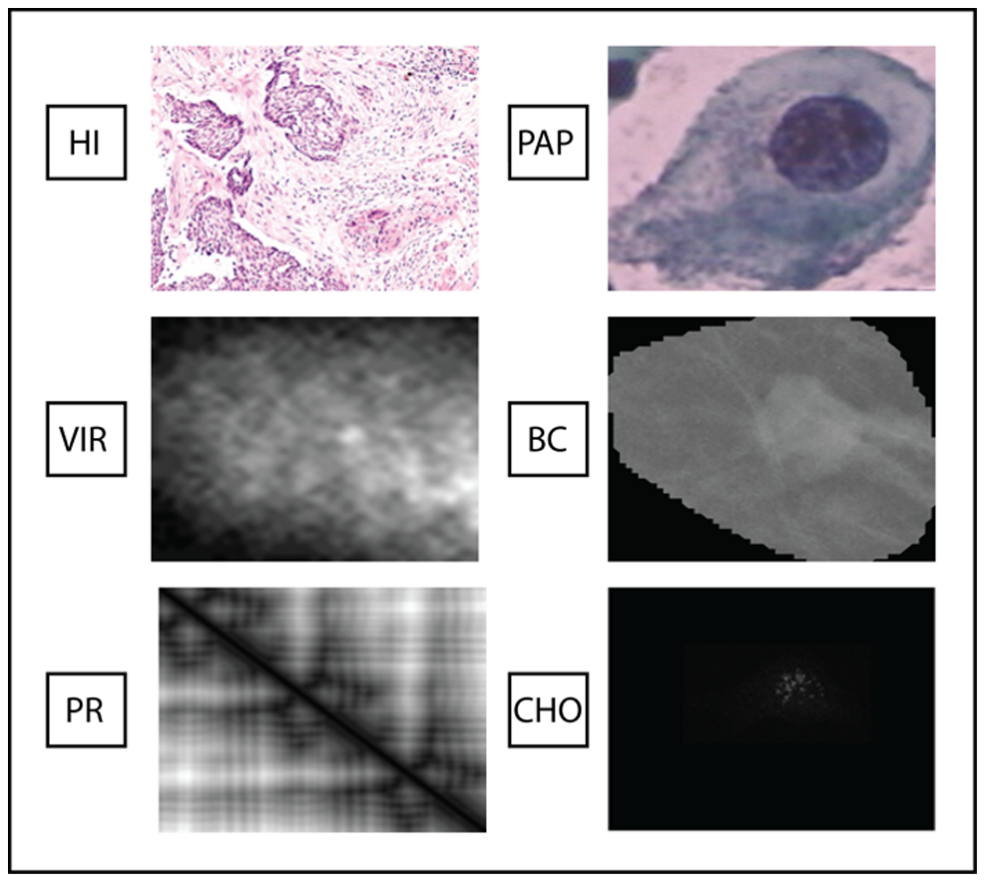
Illustration of image diversity with a sample image representative of each of the six datasets.

We validate our systems across six medical datasets to assess generality. These datasets represent different computer vision problems. As an example of image diversity, Figure 3 shows the different visual characteristics of sample images representative of our six datasets.

The datasets used for evaluating approaches are the following (notice that the RGB images are converted in gray level images):

- **PAP**: this Pap Smear dataset [55] contains images representing cells that are used in the diagnosis of cervical cancer.
- **VIR**: this dataset [56] contains images of viruses extracted using negative stain transmission electron microscopy. We use the 10-fold validation division of images shared by the authors. However, we do not exploit their mask for background subtraction. Instead, we use the entire image for extracting features as this method produced better results.
- **HI**: this Histopatology dataset [57] is composed of images from different organs that are representative of the four fundamental tissues.
- **BC**: this dataset [58] contains 273 malignant and 311 benign breast cancer images.
- **PR**: this dataset, developed by [59], contains 118 DNA-binding Proteins and 231 Non-DNA-binding proteins. Texture descriptors are extracted from the 2D distance matrix that represents each protein, and this is obtained from the 3D tertiary structure of a given protein (considering only atoms that belong to the protein backbone, see [59] for details).
- **CHO**: this cell dataset [60] that contains 327 fluorescent microscopy images taken from Chinese Hamster Ovary cells and belonging to five different classes. Images are 16 bit grayscale of size 512×382 pixels.

A descriptive summary of each dataset along with website links to each dataset is reported in Table 1.

**Table 1:** Descriptive summary of the six datasets.

Name	Abbreviation	#Classes	#Samples	Sample Size	Link
Histopatology	HI	4	2828	Various	https://www.dropbox.com/s/yli3c3tbokjdc4r/histologyDS2828.tar.gz
Pap smear	PAP	2	917	Various	https://www.dropbox.com/s/rh0hj3fntd95sbp/Carcinoma.rar
Virus types classification	VIR	15	1500	41×41	http://www.cb.uu.se/~gustaf/virustexture/
Breast cancer	BC	2	584	various	Due to their large size they are available upon request to the authors of [58] or request from “nanni at dei.unipd.it”
Protein classification	PR	2	349	various	https://www.dropbox.com/s/osvc8ab7d90nsmn/BackboneProtein.rar
Chinese Hamster Ovary	CHO	5	327	512×382	http://ome.grc.nia.nih.gov/iicbu2008/

## Experiments

The 5-fold cross-validation protocol is used for testing each texture descriptor, with the exception of the VIR dataset, for which the original testing protocol is used. The area under the ROC curve (*AUC*) is used as the performance indicator because it provides a better overview of classification results. AUC is a scalar measure that can be interpreted as the probability that the classifier will assign a higher score to a randomly picked positive sample than to a randomly picked negative sample [61]. In the multi-class problem, AUC is calculated using the one-versus-all approach (a given class is considered as “positive” and all the other classes are considered as “negative”) and the average AUC is reported.

The aim of the first experiment, see table 2, is to establish the usefulness of extracting features not only from the whole co-occurrence matrix but also from different sub-windows.

**Table 2:** Table 2. Usefulness of extracting features from the co-occurrence matrix subwindows.

*AUC*	HR	HRsub	GR	GRsub	CU	CUsub	LD	LDsub	SH	SHsub
**PAP**	89.4	**92.0**	84.1	86.0	77.8	79.0	83.7	82.6	82.5	86.6
**VIR**	95.9	**96.7**	88.8	93.4	70.9	70.0	61.3	70.7	84.6	89.9
**HI**	87.7	88.5	87.3	**88.9**	67.5	65.6	–	–	83.2	87.6
**BC**	92.7	**93.6**	84.9	91.9	83.1	85.1	61.6	85.7	88.8	91.8
**PR**	90.6	91.0	85.7	**91.7**	74.8	75.3	80.4	83.5	84.8	84.7
**CHO**	99.4	99.4	98.6	97.9	98.6	97.8	**99.6**	99.5	99.5	**99.6**
***Av***	92.6	**93.5**	88.2	91.6	78.7	78.8	–	–	87.2	90.0

Due to huge memory requirements, LD is not performed on HI dataset.

The aim of the second experiment, see table 3, is to show the performance gains that can be achieved using the multi-scale approach.

**Table 3:** Table 3. Performance gains using the multi-scale approach.

*AUC*	HRsca	GRsca	CUsca	LDsca	SHsca
**PAP**	**92.5**	85.9	79.6	82.0	86.5
**VIR**	**96.7**	94.1	69.9	72.8	92.1
**HI**	89.5	**89.6**	66.6	–	**89.6**
**BC**	**93.8**	**93.8**	85.5	85.0	92.3
**PR**	91.0	**92.7**	76.3	83.2	87.6
**CHO**	99.5	97.9	98.7	99.6	**99.8**
***Av***	**93.8**	92.3	79.4	–	91.3

Due to huge memory requirements, LD is not performed on HI dataset.

Examining Tables 2 and 3, the following conclusions can be drawn:

- It is clear that all methods improve when features are also extracted from GLCM subwindows; even the standard *HR* improves when coupled with subwindow extraction (to the best of our knowledge, this is the first paper to explore using subwindows to extract information from the co-occurrence matrix);
- Coupling approaches with the multi-scale approach further improves results;
- The novel method *GRsca* obtains a performance similar to *HRsca*;
- The ensemble proposed here, *HRsca,* outperforms the base *HR;*
- *CUsca* and *LDsca* work well on some datasets and not on others: when using these approaches, it would be desirable to test them using the training data to determine whether they are suited to a specific task. For instance, CU is more suited for binary images than for texture images. LD is not well suited for extracting the local information found in the co-occurrence matrix; nonetheless, LD is able to extract some useful features.

To statistically validate of our experiments, we used the Wilcoxon signed rank test [62]:

- *HRsca* outperforms *HR* (p-value 0.05);
- *GRsca* outperforms *GR* (p-value 0.05);
- *HRsca* and *GRsca* obtain a similar performance.

Before turning to the next set of experiments, it should be observed that the results of CU and LD highlight the difficulty of predicting which descriptors will work well for a given dataset; the best way to determine this is to use the training set of each dataset to assess performance. It is well-known in the computer vision and machine learning communities that no stand-alone approach is consistently superior to any other. The “no free lunch” theorem states that any two learning algorithms exhibit the same performance when error rates are averaged across all possible problem sets (see, e.g., [63]). In our opinion, the main value of combining different descriptors is that they offer the most feasible way of coping with the “no free lunch” theorem.

The aim of the third experiment, see table 4, is to show the performance gain that is possible by fusing different descriptors extracted from the co-occurrence matrix. The descriptors chosen for this experiment were the following:

**Table 4:** Table 4. Performance of fusion approaches.

*AUC*	HRsca	GRsca	Old	SUM2	WS2	W = 2	W = 3
**PAP**	**92.5**	85.9	**92.5**	91.5	92.0	91.8	91.9
**VIR**	**96.7**	94.1	96.4	**96.7**	**96.7**	96.5	96.6
**HI**	89.5	89.6	90.7	91.3	90.9	**92.4**	92.3
**BC**	93.8	93.8	94.9	94.6	94.5	**95.1**	95.0
**PR**	91.0	**92.7**	89.1	92.7	92.3	**92.1**	92.2
**CHO**	99.5	97.9	**99.9**	99.7	99.7	**99.9**	**99.9**
***Av***	93.8	92.3	93.9	94.4	94.4	**94.6**	**94.6**

- *Old*: the best ensemble proposed in [52] based on features extracted from co-occurrence matrix;
- *SUM2*: sum rule between *HRsca* and *GRsca.* Notice: when we combine the SVMs trained with different descriptors, the scores of each SVM are always normalized to mean 0 and standard deviation 1, before the fusion step.
- *WS2*: weighted sum rule between *HRsca* (weight 2) and *GRsca* (weight 1);
- *W = 2*: weighted sum rule between *SUM2* (weight 2) and *SHsca* (weight 1);
- *W = 3*; weighted sum rule between *SUM2* (weight 3) and *SHsca* (weight 1);

The best result was obtained by choosing *W = 3*, which confirms that all three methods (*HRsca*, *GRsca* and *SHsca*) extract different information from the co-occurrence matrix. *W = 3* outperforms *HRsca* with p-value 0.05 using Wilcoxon signed rank test.

For comparison purposes, in Table 5, we report the results of some of the best performing texture descriptors reported in the literature:

**Table 5:** Table 5. Fusion approaches.

*AUC*	HRsca	LBP	LTP	MT	W1
**PAP**	92.5	87.7	86.1	90.0	**92.6**
**VIR**	96.7	89.8	91.6	93.7	**96.5**
**HI**	89.5	92.5	92.8	93.4	**94.1**
**BC**	93.8	92.4	95.6	96.0	**97.3**
**PR**	91.0	79.8	87.8	88.4	**93.2**
**CHO**	99.5	99.9	**100**	**100**	**100**
***Av***	93.8	90.3	92.3	93.6	**95.6**

- Local binary patterns (LBP) [25];
- Local ternary patterns (LTP) [64];
- Multi-threshold local quinary coding (MT) [53].

In Table 5 we also report the following fusion method:

- W1, sum rule between *MT* and *W = 3*.

It is interesting to note that the performance of the *HRsca* method that we propose is similar to what can be achieved using *MT*. Moreover, *W1* (the ensemble of different descriptors) outperforms all the base approaches that belong to it, leading to a very high performing set of descriptors.

Finally, we perform experiments using SPIN features [64] considering the GLDM as a 3D image. This method extracts reliable features, but the performance is lower than other methods reported in this work, e.g., HRsub. Using SPIN features considering the GLDM as a 3D image obtains the following results: PAP = 77.2; BC = 71.8; PR = 84.2; CHO = 93.0).

## Conclusion

In this study we extended previous work reported in the literature on texture analysis techniques based on the co-occurrence matrix. We compared and combined different strategies for extending the texture descriptors extracted from the co-occurrence matrix. These methods were further improved by combining them with a multi-scale approach based on Gaussian filtering and by extracting features not only from the entire co-occurrence matrix but also from subwindows. Moreover, we proposed a novel set of features (Grey-level run-length features) to use with the co-occurrence matrix. We also showed that our ensemble approach improves the performance of SHAPE and standard Haralick-based features and outperforms other stand-alone approaches without ad hoc dataset parameter tuning. Our proposed system was validated across six different medical image classification problems, thereby demonstrating the generality of our approach. Our results were also compared with some state-of-the-art descriptors. For all experiments SVM was used as the base classifier.

In our opinion the most valuable result of this paper is demonstrating that it is possible to extract even more information from the co-occurrence matrix than has been extracted thus far. Our study shows that it is worthwhile exploring more techniques for deriving new descriptors from the co-occurrence matrix. Future experiments, for instance, might examine different methods for processing the image before extracting the co-occurrence matrix. We hypothesize that Gabor filters and wavelet decomposition will prove to be valuable preprocessing methods.
